# Effect of Novel Pyrrolo[3,4-*d*]pyridazinone Derivatives on Lipopolysaccharide-Induced Neuroinflammation

**DOI:** 10.3390/ijms21072575

**Published:** 2020-04-08

**Authors:** Karolina Wakulik, Benita Wiatrak, Łukasz Szczukowski, Dorota Bodetko, Marta Szandruk-Bender, Agnieszka Dobosz, Piotr Świątek, Kazimierz Gąsiorowski

**Affiliations:** 1 Department of Basic Medical Sciences, Wroclaw Medical University, 50-556 Wroclaw, Poland; karolina.wakulik@student.umed.wroc.pl (K.W.); dorota.bodetko@umed.wroc.pl (D.B.); agnieszka.dobosz@umed.wroc.pl (A.D.); kazimierz.gasiorowski@umed.wroc.pl (K.G.); 2 Department of Chemistry of Drugs, Wroclaw Medical University, 50-556 Wroclaw, Poland; lukasz.szczukowski@umed.wroc.pl (Ł.S.); piotr.swiatek@umed.wroc.pl (P.Ś.); 3 Department of Pharmacology, Wroclaw Medical University, 50-345 Wroclaw, Poland; marta.szandruk@umed.wroc.pl

**Keywords:** neuroinflammation, Alzheimer’s disease, lipopolysaccharide, LPS, pyridazinone, NSAID, cyclooxygenase inhibitor

## Abstract

Neuroinflammation is considered to be one of the potential causes for the development of neurodegenerative diseases, including Alzheimer’s disease. In this study, we evaluated the effect of four newly synthesized pyrrolo[3,4-*d*]pyridazinone derivatives on the neuron-like PC12 cells under simulated inflammation conditions by preincubation with lipopolysaccharide (LPS). Our novel derivatives are selective cyclooxygenase-2 (COX-2) inhibitors and have similar effects to nonsteroidal anti-inflammatory drugs (NSAIDs). We assessed viability (LDH assay), metabolic activity (MTT assay), DNA damage (number of double-strand breaks measured by fast halo assay), and the neuronal features of cells (average neurite length and neurite outgrowth measured spectrofluorimetrically). DCF-DA and Griess assays were also performed, which allowed determining the impact of the tested compounds on the level of oxygen free radicals and nitrites. LPS administration significantly negatively affected the results in all tests performed, and treatment with the tested derivatives in most cases significantly reduced this negative impact. Multiple-criteria decision analysis indicated that overall, the best results were observed for compounds 2a and 2b at a concentration of 10 µM. The new derivatives showed intense activity against free oxygen radicals and nitrites. Reduced reactive oxygen species level also correlated with a decrease in the number of DNA damage. The compounds improved neuronal features, such as neurite length and outgrowth, and they also increased cell viability and mitochondrial activity. Our results suggest that derivatives 2a and 2b may also act additionally on mechanisms other than 3a and 3b.

## 1. Introduction

Alzheimer’s disease (AD) is the most common form of dementia worldwide and the most frequent neurodegenerative disease in the elderly. It involves the progressive and irreversible loss of nerve cells in various brain zones, in particular, the hippocampus and cortex. The disease is manifested by dementia syndrome, cognitive impairment and problems with memory, judgment, decision making, orientation, and speech. Its cause is not exactly known, and none of the currently used treatments can undo or stop the progression of the disease, despite the impressive progress in medicine. It is estimated that more than 35.6 million people with AD live in the world, and this number will double in the next 20 years [1,2].

It is widely accepted that inflammatory reactions and oxidative stress play an important role in the pathomechanism of AD, and the main pathological determinant of this disease is the accumulation of β-amyloid (Aβ) deposits within the brain [3,4,5]. The two of the most known plaques, i.e., diffuse and mature plaques, are surrounded with foci of inflammation [6,7]. As the amount of amyloid deposits increases, the amount of microglial cells striving to cleave Aβ deposits is also increasing. The continuous activation of microglia also affects the induction of immune response receptors, including Toll-like receptors (TLRs), causing inflammation [8,9]. There is much evidence that people with Alzheimer’s disease have much microglia in their brains that causes chronic inflammation. Increased expression of proinflammatory mediators such as cytokines and chemokines (IL-1β, IL-6, TNF-α), complement components (C1q, C3a, C3b), and acute-phase proteins opsonising β-amyloid deposits are observed [10].

The brain is the organ that is most sensitive to oxidative stress due to biochemical processes and the need for nutrients. With aging, iron, copper, and zinc ions accumulate in the brain, which participates in the Fenton reaction, and as a consequence, this leads to the formation of hydroxyl radicals [11,12]. At the same time, the brain has a relatively low level of non-enzymatic and enzymatic antioxidants compared to needs, making oxidative stress conditions easy to occur, especially in the elderly. In turn, oxidative stress plays a role in the production of Aβ. Consequently, inflammation and high levels of reactive oxygen species (ROS) act as a continuous feedback loop.

There is growing recognition that the gut microbiome plays a large role in the development of neurological diseases [7,13]. The content of Gram-negative bacteria in the microbiome increases with age. Gram-negative bacteria, including *Escherichia coli*, produce a lipopolysaccharide (LPS)— endotoxin, which is a component of their outer cell membrane [14,15]. LPS stimulates the production of proinflammatory mediators and free radicals in cells. This endotoxin was detected in the brains of the elderly, but its level in AD patients was noted to be three times higher. On this basis, it is believed that *E. coli* may be associated with the development of AD [16]. It was found that LPS can participate in the formation of amyloid plaques as well as myelin injury and tau hyperphosphorylation [16]. In healthy people, LPS does not enter the brain—it is only possible with the participation of additional factors, such as hypoxia or ischemia.

LPS is also widely used in scientific studies to regulate immune mechanisms such as cytokine production [15]. Research results are available that confirm its effect on the PC12 cell line through the Toll-like receptor 4 (TLR4) [17]. Our study used LPS from *E. coli* to simulate inflammation in neuron-like PC12 cells.

Effective treatment of neuroinflammation in the brain of patients with Alzheimer’s disease may slow or stop its progression [13,18]. Nonsteroidal anti-inflammatory drugs (NSAIDs) are worldwide the most commonly used analgesic, antipyretic and anti-inflammatory medicines that act by inhibition of cyclooxygenase (COX) activity [19]. A protein involved in response to inflammation is mainly cyclooxygenase-2 (COX-2) [20]. There are also COX-1 and COX-3 isoforms, but COX-3 is thought to be not functional in humans [20,21]. COX-1 is involved in signaling pathways and tissue homeostasis and is needed for the proper functioning of the human digestive and circulatory systems. Most NSAIDs inhibit not only COX-2 but also COX-1, which causes many side effects. It has been observed that long-term use of NSAIDs (e.g., ibuprofen or naproxen) may lead to gastrointestinal ulceration [19,22].

The study examined the effect of LPS on the viability and neuronal features of differentiated neuron-like PC12 cells. Thus, it was evaluated whether this endotoxin could have a negative effect directly on neuronal cells without enhancing the inflammatory response by microglia or astrocytes. As bacterial toxins have been observed in the brains of AD patients, their direct effects on neurons cannot be excluded, which has been checked in this study.

**Figure 1:**
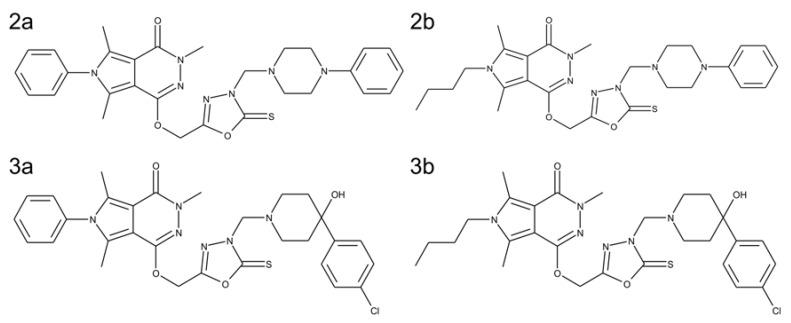
Structures of tested pyrrolo[3,4-*d*]pyridazinone derivatives: (**2a**) 3,5,7-trimethyl-6-phenyl-1-[[3-[(4-phenylpiperazin-1-yl)methyl]-2-thioxo-1,3,4-oxadiazol-5-yl] methoxy]pyrrolo[3,4-*d*]pyridazin-4-one; (**2b**) 6-butyl-3,5,7-trimethyl-1-[[3-[(4-phenylpiperazin-1-yl)methyl]-2-thioxo-1,3,4-oxadiazol-5-yl] methoxy]pyrrolo[3,4-*d*]pyridazin-4-one; (**3a**) 1-[[3-[[4-(4-chlorophenyl)-4-hydroxy-1-piperidyl]methyl]-2-thioxo-1,3,4-oxadiazol-5-yl] methoxy]-3,5,7-trimethyl-6-phenyl-pyrrolo[3,4-*d*]pyridazin-4-one; (**3b**) 6-butyl-1-[[3-[[4-(4-chlorophenyl)-4-hydroxy-1-piperidyl]methyl]-2-thioxo-1,3,4-oxadiazol-5-yl] methoxy]-3,5,7-trimethyl-pyrrolo[3,4-*d*]pyridazin-4-one.

However, the main goal of the study was to investigate the activity of novel pyrrolo[3,4-*d*]pyridazinone derivatives for potential use in neurodegenerative diseases, including Alzheimer’s disease. Literature data indicate that many pyridazinone derivatives exhibit selective inhibition of the COX-2 enzyme [19]. We have tested in our previous study some newly synthesized pyridazinone derivatives for their preferential inhibition of COX-2 over COX-1 [23], and on this basis, the four compounds were selected for further study on their effects on neuronal cell viability and neuronal properties. Structures of the selected derivatives are presented in Figure 1. We assumed that our new compounds, which are COX inhibitors, could potentially affect the regeneration of neuronal cells with damage caused by neuroinflammation simulated by preincubation with LPS. To assess the effect of our derivatives, a number of culture parameters were evaluated: viability, metabolic activity, levels of oxygen free radicals and nitric oxide, DNA damage, and neuronal properties such as the average length and outgrowth of neurites. The study allowed us to choose the most promising derivatives for further in vitro studies using cocultures and eventual in vivo testing.

## 2. Results

### 2.1. Chemistry

**Figure 2:**
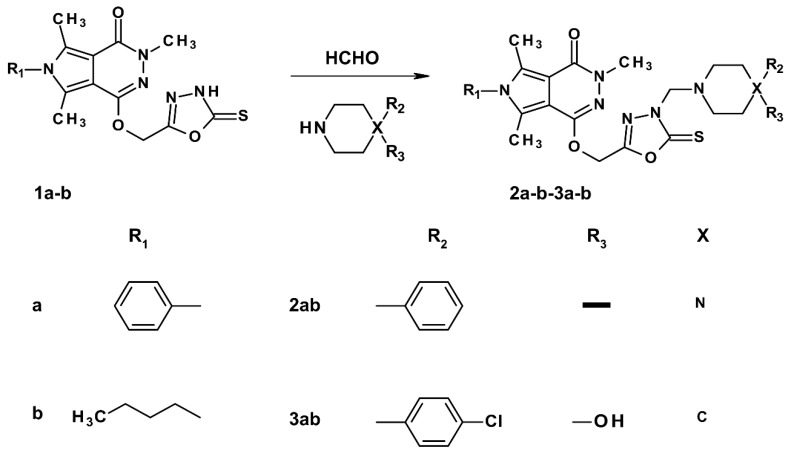
Synthesis and structures of examined derivatives of pyrrolo[3,4-*d*]pyridazinone.

The synthesis of all investigated compounds was performed according to the protocols published previously [24,25,26] and is presented in Figure 2. Analytical and spectroscopic properties of all newly obtained derivatives were in good agreement with their predicted features.

The final Mannich bases **2a**,**b-3a**,**b** were obtained in good yield via a convenient and efficient one-step reaction of **1a**-**b** with appropriate secondary amine and formaldehyde in anhydrous ethanol at room temperature. The characteristic signal in the ^1^H NMR spectrum near δ 4.98–5.10 ppm and the peak at around δ 70.01–70.42 ppm in the ^13^C NMR spectrum indicate the presence of the methylene link that is typical for Mannich bases. According to many studies, the presence of an arylpiperazinyl or arylpiperidinyl group can significantly potentiate the analgesic activity of compound [19,26,27,28,29]. Thereby, in our investigations, different amines were deliberately chosen to evaluate the influence of this pharmacophore on biological activity.

Received crude products were purified with the use of silica gel column chromatography or by crystallization from the appropriate solvent. The structures of all new compounds were determined and affirmed by spectroscopic techniques: ^1^H NMR, ^13^C NMR, MS, FTIR, elemental analysis, and based on their physicochemical features.

### 2.2. Viability and Neuronal Features of Cells

**Figure 3:**
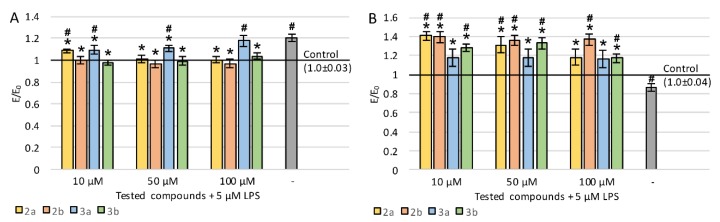
Effect of tested compounds on PC12 cells preincubated with lipopolysaccharide (LPS): (**A**) cell viability measured in LDH assay, (**B**) metabolic activity measured in MTT assay; Control—cell culture incubated without LPS and tested substances; * *p* < 0.05—significant difference compared to control preincubated with LPS; **#** *p* < 0.05—significant difference compared to the negative control without LPS.

To study the neuroregenerative effects of the tested derivatives on the viability of PC12 cells after differentiation with nerve growth factor (NGF) and exposition to LPS, lactate dehydrogenase (LDH) release was evaluated (Figure 3A). The incubation of cells with LPS resulted in a statistically significant increase in LDH leakage (20.6%). All tested compounds significantly reduced LDH release in almost the entire concentration range (except for the concentration of 100 µM of compound 3a) compared to control with LPS. For derivatives 2a (except for 10 μM), 2b, and 3b, the LDH leakage was reduced to a level similar to a negative control without LPS.

Cellular metabolic activity was evaluated by measurement of the reduction of tetrazolium salt in mitochondria in MTT assay (Figure 3B). LPS treatment resulted in a 14% decrease in mitochondrial activity. The administration of the tested derivatives (regardless of concentration) caused a large increase in metabolic activity, for derivatives 2a (except for 100 μM), 2b, and 3b to a statistically significantly higher level even compared to the control without LPS. A clear concentration dependence was observed for compound 2a—the lower the concentration, the higher the metabolic activity. Derivative 2b also showed the strongest activity at the lowest concentration, while the effect of 3a was concentration-independent, and in the case of 3b, the best result was obtained at 50 μM. In general, the greatest increase in mitochondrial activity was observed after the administration of 3a or 3b derivatives at 10 μM concentration.

**Figure 4:**
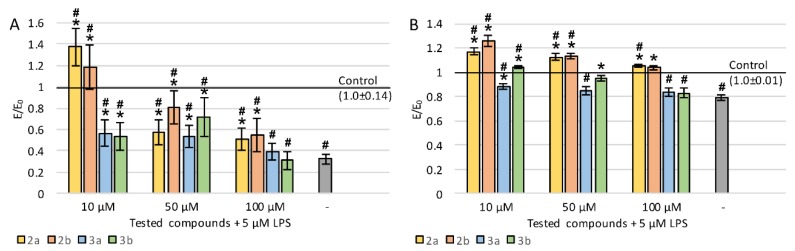
Effect of tested compounds on neurites in PC12 cells preincubated with LPS: (**A**) average length of neurites, (**B**) neurite outgrowth; Control—cell culture incubated without LPS and tested substances; * *p* < 0.05—significant difference compared to control preincubated with LPS; **#** *p* < 0.05—significant difference compared to the negative control without LPS.

To assess the effect on neuronal properties of PC12 cells, the average length of neurites was evaluated (Figure 4A). The lengths were measured for neurites that were at least twice as long as the cell body. Incubation with LPS caused a very strong decrease in neurite length—by 68%. For all derivatives (except for the concentration of 100 µM of compounds 3a and 3b), a statistically significant increase in the average length of measured neurites was observed compared to LPS-induced cells. Concentration dependence was observed for all the tested compounds—the lower the concentration, the longer the neurites. Interestingly, at a concentration of 10 µM for compounds 2a and 2b, neurites were also significantly longer compared to the negative control (without LPS).

**Figure 5:**
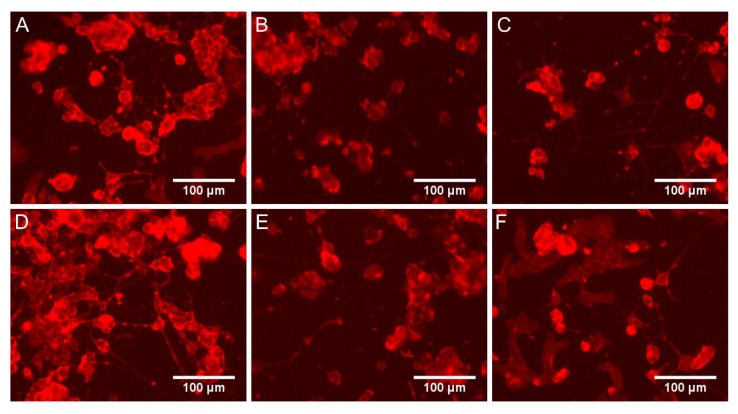
Sample microphotographs of cell cultures stained with “Neurite Outgrowth Staining Kit”: (**A**) negative control without LPS; (**B**) control with LPS; (**C**) LPS and compound 2a (50 µM); (**D**) LPS and compound 2b (50 µM); (**E**) LPS and compound 3a (50 µM); (**F**) LPS and compound 3b (50 µM).

Staining with a ready-to-use kit was also carried out which allowed evaluating neurite outgrowth in cell cultures using a spectrofluorimetric plate reader. A sample photo of a cell culture stained with a kit is shown in Figure 5. Incubation with LPS caused a decrease in neurite outgrowth by 21% (Figure 4B). Derivatives 2a and 2b significantly improved outgrowth (especially at low concentration), which reached an even higher level compared to cultures without LPS. Compound 3b at 10 and 50 µM also showed significant activity, whereas 3a was the weakest (statistically significant effect only at 10 µM). For all derivatives, the activity was stronger at low concentrations.

After incubation of tested compounds with PC12 cells, it was observed that all compounds in MTT assay in the entire range of tested concentrations caused an increase in mitochondrial activity compared to the negative control (without LPS and tested compounds). There are literature reports on the association of mitochondrial activity and neurite growth. Since the higher mitochondrial activity was observed, even compared to healthy control, correlations between MTT assay results and neurite evaluation were calculated, both in the spectrofluorimetric assay and the measurement of the average length of neurites (Table 1). For all derivatives tested, all correlations between the MTT assay results and average neurite length, as well as neurite outgrowth measured spectrofluorimetrically were strongly positive and statistically significant.

### 2.3. ROS, Nitric Oxide, and DNA Damage

**Figure 6:**
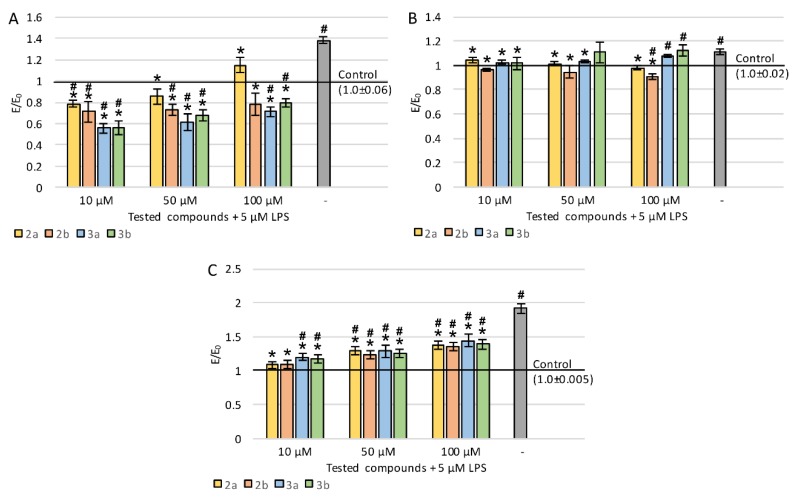
The effect of tested compounds on PC12 cell culture incubated with LPS: (**A**) DCF-DA assay, (**B**) Griess assay, (**C**) fast halo assay; Control—cell culture incubated without LPS and tested substances; * *p* < 0.05—significant difference compared to control preincubated with LPS; **#** *p* < 0.05—significant difference compared to the negative control without LPS.

Intracellular accumulation of ROS was measured using the DCF-DA assay (Figure 6A). An almost 40% increase in free oxygen radicals was observed after the incubation of cell cultures with LPS compared to the negative control. The effect of the application of each tested pyridazinone derivative showed concentration dependence. The lower the concentrations of the tested compounds, the higher their ability to reduce the ROS level. Compounds 3a and 3b showed the strongest antiradical activity. In the entire concentration range of all compounds tested, the effect was statistically significant. It should be noted that in most cases (except for the concentrations of 50 and 100 µM of compound 2a, and 100 µM of compound 2b), the level of oxygen free radicals was significantly lower than in control without LPS.

**Figure 7:**
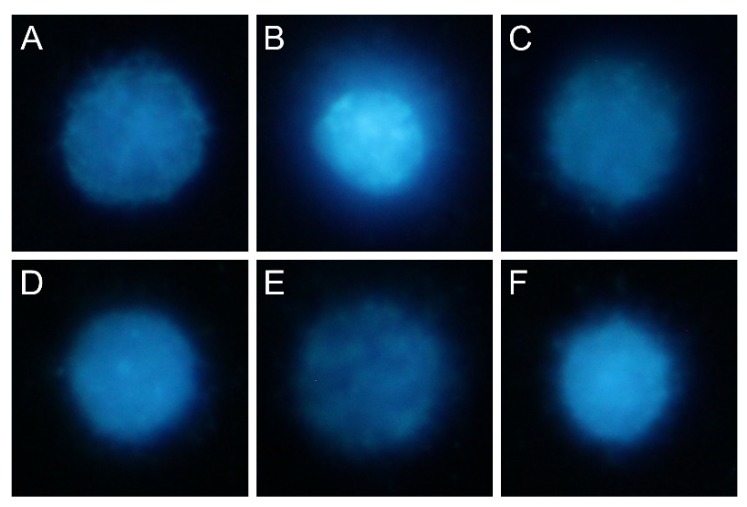
Microphotographs of a nuclear halo: (**A**) sample cell incubated without LPS and without compounds (negative control); (**B**) sample cell incubated only with LPS; (**C**) sample cell incubated with LPS and compound 2a (10 µM); (**D**) LPS and compound 2b (10 µM); (**E**) LPS and compound 3a (10 µM); (**F**) LPS and compound 3b (10 µM).

High levels of oxygen and nitrogen free radicals can lead to DNA damage, including double-strand breaks (DSBs). The number of DSBs was assessed in the fast halo assay (FHA) by measuring the size of the nuclear halo (chromatin dispersion). Results are presented in Figure 6C and sample photos with the halo are shown in Figure 7. In LPS-induced cells, the DSB number was almost twice as high compared to the negative control. Activity dependence on concentration has been demonstrated for all derivatives tested—the lower the concentration, stronger the regeneration of DNA damage occurred. In all cases, the regenerative effect was statistically significant. For derivatives 2a and 2b at a concentration of 10 µM, DSBs decreased even to a level similar to the negative control without LPS.

Correlation coefficients between DNA damage and ROS or nitric oxide (NO) levels were determined (Table 2). In all derivatives tested, correlations between the results of DCF-DA and FHA assays (ROS level versus DNA damage) were strongly positive and statistically significant. Correlations between DNA damage and nitrite ion level for compounds 2a and 2b were strongly negative, whereas, for the compounds 3a and 3b, they were strongly positive. Along with the decrease in the ROS level (and NO level for the compounds 3a and 3b), a decrease in the level of DNA damage was observed, which suggests the involvement of free radical mechanisms in the formation of DNA damage in PC12 cells (as a result of LPS activity).

### 2.4. Multiple-Criteria Decision Analysis

**Figure 8:**
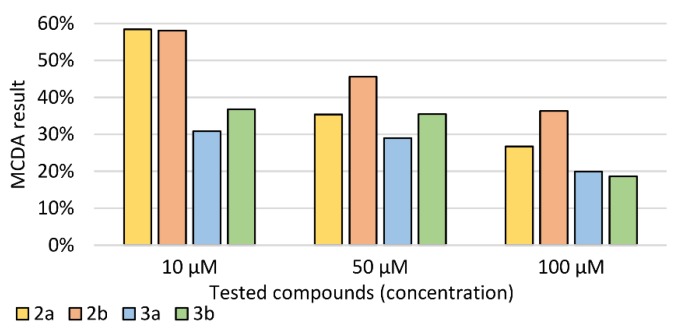
Multiple-criteria decision analysis (MCDA) of the effect of the tested pyridazinone derivatives; MCDA was calculated based on E/E_LPS_ ratios determined in individual assays, where E is the sample result, and E_LPS_ is the result in control preincubated with LPS.

The obtained results of all assays performed for four newly synthesised pyridazinone derivatives were analyzed using multiple-criteria decision analysis (MCDA). The results of MCDA (Figure 8) showed that in the tested concentration range, each compound had a beneficial neuroregenerative effect on PC12 cells previously incubated with LPS. The strongest overall effect was found for derivatives 2a and 2b at a concentration of 10 µM.

## 3. Discussion

When looking for medicines for Alzheimer’s disease, nowadays, special attention is paid to nonsteroidal anti-inflammatory drugs (NSAIDs) [22]. The mechanism of NSAIDs may exert a protective effect on neuronal cells by inhibiting cyclooxygenase (COX) activity, especially COX-2, whose expression is strongly induced by inflammatory signals [19]. Several papers describe a relationship between long-term use of NSAIDs and a reduced risk of cognitive impairment, as well as a slower decline in cognitive function [18,30]. However, the mechanism of protective action has not been fully elucidated. Both COX-1 and COX-2 are physiologically expressed naturally in a healthy brain, but elevated COX-2 levels have been found in the early stages of AD [31,32]. The basis of NSAIDs administration is the inhibition of COX activity, but another mechanism responsible for their effectiveness in reducing the risk of developing dementia cannot be excluded [33]. Some NSAIDs have been shown to be potential inhibitors of β-amyloid fibril formation. They can also activate the expression of other proteins that prevent Aβ aggregation or interact via peroxisome proliferator-activated receptors (PPARs) [18,33,34]. It has also been found that these drugs may affect the processing of amyloid precursor protein (APP) by β- and γ-secretases [35]. The current state of knowledge is insufficient and requires further research on the impact of specific NSAID mechanisms on neuroinflammation in dementia, including Alzheimer’s disease.

In the material collected posthumously from the brain of Alzheimer’s patients, bacterial toxins were observed. Bacterial molecules such as genetic material, cell wall fragments, including peptidoglycans, flagellins, and lipopolysaccharide (which has a strong proinflammatory effect) have been shown to pass through blood–brain barrier under pathological conditions [16,36]. Moreover, LPS, similar to bacterial amyloids, participates in the formation of senile plaques in the AD brain [7,16,37,38,39].

It is well known that LPS causes inflammation by activating microglia and astrocytes [15]. However, due to the presence of this endotoxin in the brains of AD patients, its direct effect on neuronal cells is not excluded. This direct effect of LPS on the viability and neuronal characteristics of neuron-like PC12 cells was used in this study. An obvious further direction of research into the activity of new compounds is the study involving immune cells, e.g., through the use of co-cultures of microglia and neuronal cells. The involvement of immune cells would additionally investigate the indirect effects of LPS on neuronal cells, primarily through the proinflammatory cytokines IL-1β and IL-6 [9].

In our study on PC12 cells, after LPS administration, we observed an increase in ROS and reactive nitrogen species (RNS) levels as well as the number of double-stranded DNA breaks. Incubation with LPS also significantly reduced the viability measured by the LDH release, cellular metabolic activity, and their neuronal features (length and outgrowth of neurites). These results confirm that LPS also has a direct effect on neuron-like cells (at least *in vitro*).

LPS is thought to affect PC12 cell death through three effects: proinflammatory, proapoptotic, and oxidative stress. From several studies, it is known that the proinflammatory effect of LPS on PC12 cells is based on activation of the TLR4 (Toll-like receptor 4). Activation of the TLR4 receptor induces NF-κB transcription factor, which promotes the expression of proinflammatory cytokines and chemokines [9,14,17,40]. The treatment of PC12 cells with LPS at a concentration of 1 mg/mL for 18 h resulted in an approximately six-fold increase in NF-κB compared to the control [20]. Consecutively, an increased level of proinflammatory cytokines (IL-1β, IL-6, TNF-α) promotes the expression of COX-2 [41,42]. Cells exposed to LPS could also initiate an apoptotic program and release the HMGB1 protein outside the cells, which binds to the RAGE, TLR2, and TLR4 receptors on the surface of adjacent cells, inducing an inflammatory reaction in them through NF-κB stimulation [3,41,42,43]. It is also known that incubation of PC12 cells with LPS leads to cell death by apoptosis, due to increased level of a pro-apoptotic Bax protein and reduction of anti-apoptotic Bcl-2 [17,44].

The administration of novel pyrrolo[3,4-*d*]pyridazinone derivatives reduced the adverse effects of incubation with LPS according to all assays performed. From another study of ours, we know that the tested compounds are selective COX-2 inhibitors [23]. Other researchers have also indicated the selective COX-2 activity of many pyridazinone derivatives [19]. Based on the literature data, one can attempt to explain the possible mechanisms of action of the derivatives studied. By inhibiting COX-2, there was probably a decrease in NF-κB expression, followed by a decrease in proinflammatory cytokine production in cell cultures. Tested derivatives may also have an effect on reducing the level of the HMGB1 protein, which may have expanded the inflammatory response. Yang et al. showed that aspirin, which is a nonselective NSAID drug, works by inhibiting COX enzymes, but its action in the aspect of tumor cell migration and cancer invasiveness can be linked to its inhibitory activity on the HMGB1 protein and not on the COX enzymes [45]. Thus, it cannot be excluded that also the tested pyridazinone derivatives act on the HMGB1 protein independently to their influence on the COX-2 enzyme.

The data presented in our paper show that incubation of PC12 cells with LPS leads to an increase of ROS and RNS levels. The addition of the tested derivatives markedly reduced oxidative stress and also slightly decreased the cellular content of nitrogen radicals, although this decrease was not statistically significant. For this reason, it should be taken into account that the tested compounds also acted based on other mechanisms related to the reduction of oxidative and nitrosative stress, such as pathways associated with the p53 protein [46].

Administration of the tested pyridazinone derivatives significantly reduced DNA damage caused by the preincubation of PC12 cells with LPS, and this effect of the tested compounds was strongly correlated with their impact on lowering of oxidative stress.

It should be noted that treatment with novel derivatives resulted in an increase in metabolic activity and oxidative stress, even when compared to a control that was not preincubated with LPS. The MTT assay is commonly used as an indirect indicator of cell viability, but interpreting its results, one should take into account that many factors can alter the metabolic activity, such as an increase of the number of mitochondria and also their reorganization in developing neurons [47,48].

The impact of the tested compounds on neurite length and outgrowth in PC12 cells preincubated with LPS differed markedly, especially in a lower concentration of the tested pyridazinone derivatives (10 μM). For example, in the case of compounds 2a and 2b, neurite length was about 40% and 20% higher (respectively) compared to control cultures incubated with LPS without tested compounds, whereas for compounds 3a and 3b, neurite length was 40% smaller than in control cultures. This highly beneficial effect of derivatives 2a and 2b at low concentrations on neuronal properties of PC12 cells cannot be explained only by a reduction of ROS level—in this respect, they were weaker than 3a or 3b. The reason for the observed differences in the impact of the tested compounds may be a different effect on the metabolic and phenotypic reprogramming in differentiating neurons. In the study on primary neuronal cell cultures, during differentiation, a substantial increase in mitochondrial mass was observed, as well as changes in their morphology and function [49]. At the same time, an increase in glucose metabolism was observed, which was associated with an increase in uptake and increased expression of GLUT3 mRNA. The authors stated that the PI3K/Akt/mTOR pathway is responsible for the regulation of energy metabolism in neurons [49]. Since the increase in metabolic activity of mitochondria is considered to be associated with increased neuronal differentiation, it is planned to study the effect of tested compounds on glucose metabolism in PC12 cell cultures in order to determine the causes of the observed differences in the effect of the tested compounds on the neuronal properties of these cells.

In conclusion, the newly synthesised pyrrolo[3,4-*d*]pyridazinone derivatives markedly decreased intracellular ROS level in PC12 cells in which inflammation was simulated by the administration of LPS. Reduced cellular ROS level strongly correlated with a decrease in DNA damage. The tested compounds improved some phenotypic features of PC12 neuronal differentiation, such as neurite length and outgrowth, and they also increased cell viability and mitochondrial activity. Further research is necessary to clarify the mechanism of action of the compounds tested, as well as to explain the reasons for the significant differences in their effect on PC12 neuronal properties—compounds 2a and 2b appear to act additionally also on mechanisms other than 3a and 3b.

## 4. Materials and Methods

### 4.1. Cell Line

The study was carried out using the PC12 cell line obtained from the ATCC (USA). These cells, after differentiation with nerve growth factor (NGF), stop proliferating and become similar to adult sympathetic cells [50,51]. Therefore, despite the neoplastic origin from rat adrenal pheochromocytoma, they are often used in neuroscience studies.

Cells were cultured at 37 °C in a humidified 5% CO_2_/95% air atmosphere incubator and passaged twice weekly by transferring cells to the tube and centrifuging for 5 min at 1000× *g*. After this time, the supernatant was removed, and fresh medium was added. The cell clumps were broken by squeezing through a 0.7 mm needle twice.

### 4.2. Cell Culture Media

Two types of medium were used in the study: primary medium and differentiation medium. The cells were cultivated in primary medium RPMI 1640 supplemented with 10% donor horse serum (DHS), 5% FBS fetal bovine serum (FBS), 2 mM L-glutamine, 1.25 µg/mL amphotericin B, and 100 µg/mL gentamicin. Differentiation medium was also RPMI 1640 but with reduced serum (only 1% DHS) and the addition of 100 ng/mL nerve growth factor (NGF). Prepared culture media were stored at 4–8 °C for up to one month.

### 4.3. Tested Compounds

Tested pyrrolo[3,4-*d*]pyridazinone derivatives were obtained from the Department of Chemistry of Drugs at Wroclaw Medical University. The cytotoxicity of tested compounds has already been assessed on the NHDF (normal human dermal fibroblasts) cell line [23]. After culturing NHDF cells for 24 h with the tested compounds, an SRB assay was performed. Based on the results obtained, IC_50_ values for individual derivatives were determined, which were 414.1, 191.7, 169.2, and 377.3 μM for compounds 2a, 2b, 3a, and 3b, respectively. A non-toxic concentration range of 10–100 µM was selected for this study on neuron-like PC12 cells. Tested compounds were dissolved in DMSO to a stock concentration of 10 mM.

LPS (Sigma-Aldrich, Saint Louis, USA, cat. no. L2630) from Escherichia coli was dissolved in distilled water to a stock concentration of 1 mM. For the experiment, LPS was used in the concentration of 50 µM.

All prepared stock solutions were stored at –20 °C for up to 6 months. To achieve the final concentration, all compounds were dissolved in the primary medium.

### 4.4. Modification of the Surface of Culture Plates

The surface of the culture plate wells was modified to allow adhesion of PC12 cells (they do not adhere to plastic surfaces) [51]. To this end, collagen was used, which occurs naturally in the extracellular matrix (ECM). Type I collagen was purchased from Sigma-Aldrich and dissolved in 0.1 M acetic acid to a stock concentration of 0.1% (w/v). The solution prepared in this way was stored at –20 °C for up to 6 months. Before the experiment, a working collagen solution was prepared by diluting the stock solution in distilled water 10 times to a final concentration of 0.01% (w/v). The solution was added to the wells in a volume needed to cover the surface of the wells. The plates were placed at 4–8 °C overnight. The next day, the remaining collagen solution was removed, and the plate was washed three times with PBS. Collagen-coated plates were stored at 4 °C for up to one month. Before using them in the experiments, they were irradiated with UV for 30 min.

### 4.5. Experimental Design

Cells were seeded at a density of 10,000 cells/well in multiwell culture plates, except for FHA assay, in which cells were seeded at 25,000 cells/well. After the cells adhere to the collagen-coated surface (24 h after seeding), the primary medium was removed, and the differentiation medium with NGF was added for 72 h to induce neurite formation. After this time, inflammation conditions were simulated by exposing PC12 cells to LPS at a concentration of 50 µM for 24 h. Then, LPS was removed, cultures were washed with PBS, and tested compounds were added for 24 h to assess whether they exert neuroregenerative effects on LPS-induced cells.

Two controls were used in the study. Negative control was used as a reference, which was a cell culture incubated in the primary medium without LPS and without tested compounds. The second control was a culture with LPS but without tested compounds.

Cell viability was measured by LDH release, metabolic activity in MTT assay, oxygen free radicals, and nitric oxide levels in DCF-DA and Griess assays, respectively. The number of DNA double-strand breaks (DSBs) was assessed by FHA assay. Neurite outgrowth was analyzed using a ready-to-use kit, and the average neurite length was measured using microscopic images and ImageJ software.

### 4.6. LDH Assay

Cell viability was evaluated using the colorimetric “Pierce LDH Cytotoxicity Assay Kit” (Thermo Scientific, Rockford, USA, cat. no. 88954) according to the manufacturer’s instructions. The cytoplasmic enzyme lactate dehydrogenase is released into the cell culture supernatant upon damage of the plasma membrane. After incubation with tested compounds, the supernatant was aspirated and transferred to another plate. Subsequently, the reaction mixture was added to plates with the collected supernatant, and the plates were left at room temperature (RT) for 30 min (protected from light) to allow the reaction to proceed. At the end of the 30 min period, a stop solution was added to terminate the reaction, and the measurement of absorbance was performed at 490 nm and 680 nm using a Varioskan LUX microplate reader (Thermo Scientific, USA).

### 4.7. MTT Assay

The MTT assay was used to measure the effect of tested compounds on the metabolic activity of PC12 cells. After incubation with LPS and tested compound, the supernatant was removed, 1 mg/mL MTT solution in MEM was added to each well, and plates were incubated for 2 h at 37 °C. After incubation time, the medium was removed. Formazan crystals were dissolved in 100 µl of isopropanol for 30 min, and absorbance was measured at 570 nm using Varioskan LUX microplate reader (Thermo Scientific, USA).

### 4.8. Level of Reactive Oxygen Species

The level of reactive oxygen species (ROS) was measured using the DCF-DA assay. After 1 h of incubation with compounds, the culture medium was removed, and cells were washed with PBS. Then, 25 µM of DCF-DA solution in MEM without serum and phenol red was added for 1 h at 37 °C. The ROS level was determined fluorimetrically with excitation at 485 nm and emission at 535 nm using a Varioskan LUX microplate reader (Thermo Scientific, USA).

### 4.9. Griess Assay

A standard procedure of Griess assay was used to determine nitrite ions production in PC12 cell cultures. After incubation with tested compounds for 1 h, 50 µl of supernatant was transferred into a new plate, and 50 µl of Griess reagent (1:1 mixture (v/v) of 1% sulfanilamide in 5% phosphoric acid and 0.1% N-(1-Naphthyl)ethylenediamine dihydrochloride) was added. The plate thus prepared was left for 20 min in the dark at RT. Nitrite level was measured at 548 nm using a Varioskan LUX microplate reader (Thermo Scientific, USA).

### 4.10. Fast Halo Assay

Double-strand breaks (DSBs) in DNA were assessed using fast halo assay (FHA). After 24 h of incubation with tested compounds according to procedures described earlier, cells were detached from the surface of the plate with a TrypLE solution for 3 min. The cells were transferred to the tube, and TrypLE solution was inactivated by adding the same volume of the medium. Then, the cells were centrifuged at 1000 × g for 5 min. The supernatant was removed, and the cell pellet was washed in PBS and centrifuged again under the same conditions. The next step was to remove the supernatant and suspend the cells at a density of 1000 cells/µl in PBS with Ca^2+^ and Mg^2+^. The tubes with the cells were placed in a water bath. Then, 120 ul of 1.25% low melting agarose in PBS was added to the cell suspension, and the mixture was immediately squeezed between a slide coated with an agarose (high melting point) and a coverslip. After 10 min of gel formation on the cooling block, the coverslips were removed, and slides were placed in the lysis buffer overnight. The next day, the slides were transferred into alkaline solution (pH = 13.0) for 30 min and then washed twice for 5 min in neutralizing buffer. Slides were stained using 5 µM DAPI for 20 min and examined immediately under a fluorescence microscope. The pictures were taken, and then the ratio of cell nucleus diameter to halo diameter was analyzed (which is a measure of DNA damage).

### 4.11. Neurite Outgrowth

Neurite outgrowth is one of the most important parameters in neuronal cell cultures and provides an easy way to determine the effect of the tested substance on this type of cell culture. In the experiment, a ready-to-use “Neurite Outgrowth Staining Kit” (Thermo Fisher Scientific, USA, cat. no. A15001) was used. The supernatant was removed, and the cells were washed with PBS. Then, the staining solution prepared according to the manufacturer’s instructions was added, and plates were incubated for 20 min at 37 °C. Cells were then rewashed with PBS, and background suppression dye was administered. A spectrofluorimetric measurement was taken using a Varioskan LUX microplate reader (Thermo Scientific, USA) at an excitation wavelength of 485 nm and emission 535 nm. Besides, cultures were also evaluated under an EVOS microscope with a fluorescence filter.

### 4.12. Length of Neurites

The length of neurites was analyzed based on microscopic images using the ImageJ software. Average lengths were measured for 50 cells in each of 5 independent replicates. Only cells whose neurite length was twice as large as the body diameter were evaluated.

### 4.13. Statistical Analysis

All results are presented as mean ± SEM (standard error of the mean) relative to the control (E/E_0_), where E is the culture with the tested substance and E_0_ is the negative control (without LPS and tested compounds). The E/E_0_ ratio was also calculated for the control culture with LPS given. In this way, graphs show the activity of the tested pyrrolo[3,4-*d*]pyridazinone derivatives compared to normal (healthy) cultures as well as cultures of LPS-induced cells. Statistical significance was calculated compared to the control with LPS.

Due to the lack of a normal distribution, the non-parametric Kruskal–Wallis test was used (with appropriate post-hoc tests). In all assays, *p* < 0.05 was used as the significance level. In order to compare the activity of the tested compounds, multiple-criteria decision analysis (MCDA) was carried out using a weighted sum model (WSM). The weights were chosen according to the meaning of each biological assay. Pearson correlation coefficients were calculated to show the relationship between DNA double-strand breaks and free radical levels.

## Figures and Tables

**Table 1:** Pearson correlation coefficients between the mitochondrial activity and the average length of neurites or neurite outgrowth measured spectrofluorimetrically.

Compound	Length of Neurites *vs.* Mitochondrial Activity	Neurite Outgrowth *vs.* Mitochondrial Activity
2a	0.864	0.993
2b	0.768	0.741
3a	0.987	0.769
3b	0.991	0.741

**Table 2:** Pearson correlation coefficients between DNA damage and reactive oxygen species (ROS) or NO levels.

Compound	ROS Level *vs.* DNA Damage	NO Level *vs.* DNA Damage
2a	0.856	−0.970
2b	0.927	−0.950
3a	1.000	0.979
3b	0.987	0.879
